# A non-intrusive reduced-order model for finite element analysis of implant positioning in total hip replacements

**DOI:** 10.1007/s10237-024-01903-w

**Published:** 2024-11-13

**Authors:** Marlis Reiber, Fynn Bensel, Zhibao Zheng, Udo Nackenhorst

**Affiliations:** 1 Institute of Mechanics and Computational Mechanics (IBNM), Leibniz University Hannover, Appelstraße 9a, 30167 Hannover, Germany; 2 TRR 298: Safety Integrated and Infection Reactive Implants (SIIRI), Hannover Medical School, Carl-Neuberg-Straße 1, 30625 Hannover, Germany; 3 International Research Training Group (IRTG) 2657, Leibniz University Hannover, Appelstraße 11/11 a, 30167 Hannover, Germany

**Keywords:** Bone remodelling, Patient-specific simulation, Parametric surrogate model, Proper orthogonal decomposition, Radial basis function interpolation

## Abstract

Sophisticated high-fidelity simulations can predict bone mass density (BMD) changes around a hip implant after implantation. However, these models currently have high computational demands, rendering them impractical for clinical settings. Model order reduction techniques offer a remedy by enabling fast evaluations. In this work, a non-intrusive reduced-order model, combining proper orthogonal decomposition with radial basis function interpolation (POD-RBF), is established to predict BMD distributions for varying implant positions. A parameterised finite element mesh is morphed using Laplace’s equation, which eliminates tedious remeshing and projection of the BMD results on a common mesh in the offline stage. In the online stage, the surrogate model can predict BMD distributions for new implant positions and the results are visualised on the parameterised reference mesh. The computational time for evaluating the final BMD distribution around a new implant position is reduced from minutes to milliseconds by the surrogate model compared to the high-fidelity model. The snapshot data, the surrogate model parameters and the accuracy of the surrogate model are analysed. The presented non-intrusive surrogate model paves the way for on-the-fly evaluations in clinical practice, offering a promising tool for planning and monitoring of total hip replacements.

## Introduction

Total hip replacement (THR) or total hip arthroplasty (THA) is a successful surgery for the treatment of osteoarthritis (Learmonth et al. 2007; Ferguson et al. 2018). Crucial factors for the success of the implantation are the positioning and size of the implant. Oversizing of the implant can cause fractures, whereas undersizing of the implant may lead to poor osseointegration resulting in potential loosening. Furthermore, if the press-fit of the implant is too tight, it may lead to a decrease in bone mass density (BMD) due to stress shielding (Floerkemeier 2021).

Currently, physicians mainly rely on medical imaging and empirical knowledge to plan hip implant surgeries. Digital preoperative planning tools can assist surgeons in patient-specific planning (Floerkemeier 2021). For routine monitoring of implants after the implantation, surgeons rely on X-ray images (Roth et al. 2012; Miller 2012). First developments employ machine and deep learning approaches for automated planning based on radiographs or to predict the outcome of THA procedures (Kim et al. 2022; Helm et al. 2020).

High-fidelity computational models are already available to predict the short- and long-term stability of hip implants, considering patients’ individual conditions, e.g. femur geometry or implant position (Nackenhorst 2018; Sun et al. 2024). The long-term stability of implants can be assessed by simulating bone remodelling, a process governed by Wolff’s law, which states that bones adapt their structure to altered loading conditions (Wolff 1892). The finite element method (FEM) can be used to simulate the bone remodelling process. Starting from purely phenomenological models (Carter et al. 1989; Beaupré et al. 1990; Huiskes et al. 1992; Weinans et al. 1992; Nackenhorst 1997), more sophisticated models have been developed, including anisotropic behaviour or multiscale models (Doblaré and García 2002; Krstin et al. 2000; Webster and Müller 2011).

However, due to their complexity, the available high-fidelity models are not yet feasible for application in clinical practice (Nackenhorst 2018; Webster and Müller 2011). Current research and applications are increasingly focusing on patient-specific simulations. Optimising the implantation for an individual patient requires assessing the success of various implant positions. The execution of a high-fidelity simulation for each potential implant position would consume extensive time and computational resources. Consequently, this time delay could negatively impact the overall planning and treatment process and thereby impact the efficiency and effectiveness of patient care.

Reduced-order models (ROMs) or surrogate models offer the possibility to decrease the computational effort while maintaining a certain level of accuracy. The reduced complexity of these models facilitates the application of computational simulations in daily clinical practice, as predictions of the BMD distributions for different implant positions are available almost immediately. Various ROMs or surrogate models have been successfully applied in biomechanics and for fast patient-specific simulations, e.g. analysing the influence of the shape of blood vessels on the haemodynamics (Niroomandi et al. 2012; Manzoni et al. 2012; Biancolini et al. 2020; Chinesta et al. 2023).

Commonly used reduction methods include reduced basis methods, where the high-fidelity problem is projected onto a reduced-dimensional subspace represented by certain basis functions. The set-up of the reduced model is divided into an offline and an online stage. During the offline stage, several computationally expensive high-fidelity simulations are performed to generate the so-called snapshots, from which the reduced basis are derived. Proper orthogonal decomposition (POD) is the most prominent method for constructing a reduced basis model. The POD method applies to linear, nonlinear and parametric-varying systems. During the online stage, a linear combination of the reduced basis and specific weights is used to efficiently approximate solutions for new parameter combinations (Quarteroni et al. 2015; Chinesta et al. 2017).

Intrusive and non-intrusive methods exist for the calculation of the weights. Intrusive methods work directly with the governing equations of the model and require the solution of a reduced-order system. In cases where an affine decomposition of the differential operator is not initially available, methods like the empirical interpolation method (EIM) or the discrete empirical interpolation method (DEIM) can be employed to recover this decomposability (Barrault et al. 2004; Chaturantabut and Sorensen 2010). In contrast, non-intrusive methods work with existing high-fidelity solvers and thus offer the possibility to use commercial FEM software (Quarteroni et al. 2015). Different metamodelling or surrogate modelling techniques can be applied to calculate the weights, e.g. polynomials, radial basis functions (RBF), kriging or neural networks (Forrester et al. 2008; Kianifar and Campean 2020).

RBFs are used for interpolating multidimensional scattered data (Narcowich and Ward 2004; Hardy 1971), making them suitable for interpolating the weights of the surrogate model. A combined POD-RBF approach has been efficiently applied in the fields of, e.g. sheet metal forming (Dang et al. 2017), shallow water equations (Dutta et al. 2021) and nonlinear magnetostatics (Henneron et al. 2019) but also in biomechanics (Girfoglio et al. 2022; Balzotti et al. 2024).

As stated above, the position of the hip implant is a critical factor for the success of the THA. Different approaches exist to create an FEM mesh with a varying implant position.

The most commonly used approach is the geometry-based model generation, where a computer-aided design (CAD) model is adapted and converted into a new FEM mesh afterwards. For example, Bittens (2020) used this approach to create FEM meshes with varying implant positions and suggested a non-intrusive surrogate model for implant positioning based on an adaptive-sparse grid collocation method.

A different approach is mesh morphing techniques, which morph a reference mesh for a new implant position using methods such as RBFs, Laplace’s equation or linear elastic equations (Biancolini 2017; Grassi et al. 2011; Zheng et al. 2023; Bah et al. 2009). Bah et al. (2009) used the linear elasticity equations, whereas Zheng et al. (2023) used Laplace’s equation to change the hip implant position of a reference mesh. Morphing techniques work directly on a reference mesh and thus offer the advantage that tedious remeshing can be avoided. Further, the total number of degrees of freedom (DOFs) and the node numbering are maintained, facilitating the application of model order reduction (MOR) methods.

***Scope of the current work***

In this work, a POD-RBF approach is applied to construct a non-intrusive surrogate model to assess BMD distributions in a femur for different implant positions. The aim is to develop an efficient and accurate computational tool, which can be integrated into the clinical workflow to optimise the implantation outcome for each individual patient.

A mesh morphing approach is used on a parameterised FEM mesh to avoid tedious remeshing in the offline stage. Due to the non-intrusive surrogate model, a commercial FEM code can be used. Consequently, bone remodelling simulations using the commercial software Abaqus are performed. As the mesh morphing approach preserves the total number of DOFs and the node numbering, the resulting BMD distributions are directly stored in the snapshot matrix and the projection of the results to a common mesh is avoided. By avoiding remeshing and projecting the results, the computational effort in the offline stage is reduced. Similarly, in the online phase, the computational effort is further reduced as the same parameterised reference mesh is used to visualise the results, avoiding the need for reprojection. Thus, the surrogate model can be used to predict the BMD distribution for a new implant position efficiently.

The remainder of the article is structured as follows: in Sect. 2, the high-fidelity model, including the reference FEM model, the mesh morphing technique as well as the bone remodelling material model and its numerical treatment are described. In Sect. 3, the set-up of the POD-RBF surrogate model is explained. The results of the sensitivity analysis and the surrogate model are presented in Sect. 4 and discussed in Sect. 5. Finally, conclusions are drawn in Sect. 6.

## The high-fidelity model

The reference FEM model is described in Sect. 2.1 and the mesh morphing of this reference model is explained in Sect. 2.2. Further, the material model for the bone remodelling algorithm and its numerical treatment are detailed in Sect. 2.3.

### Reference finite element model

**Fig. 1:**
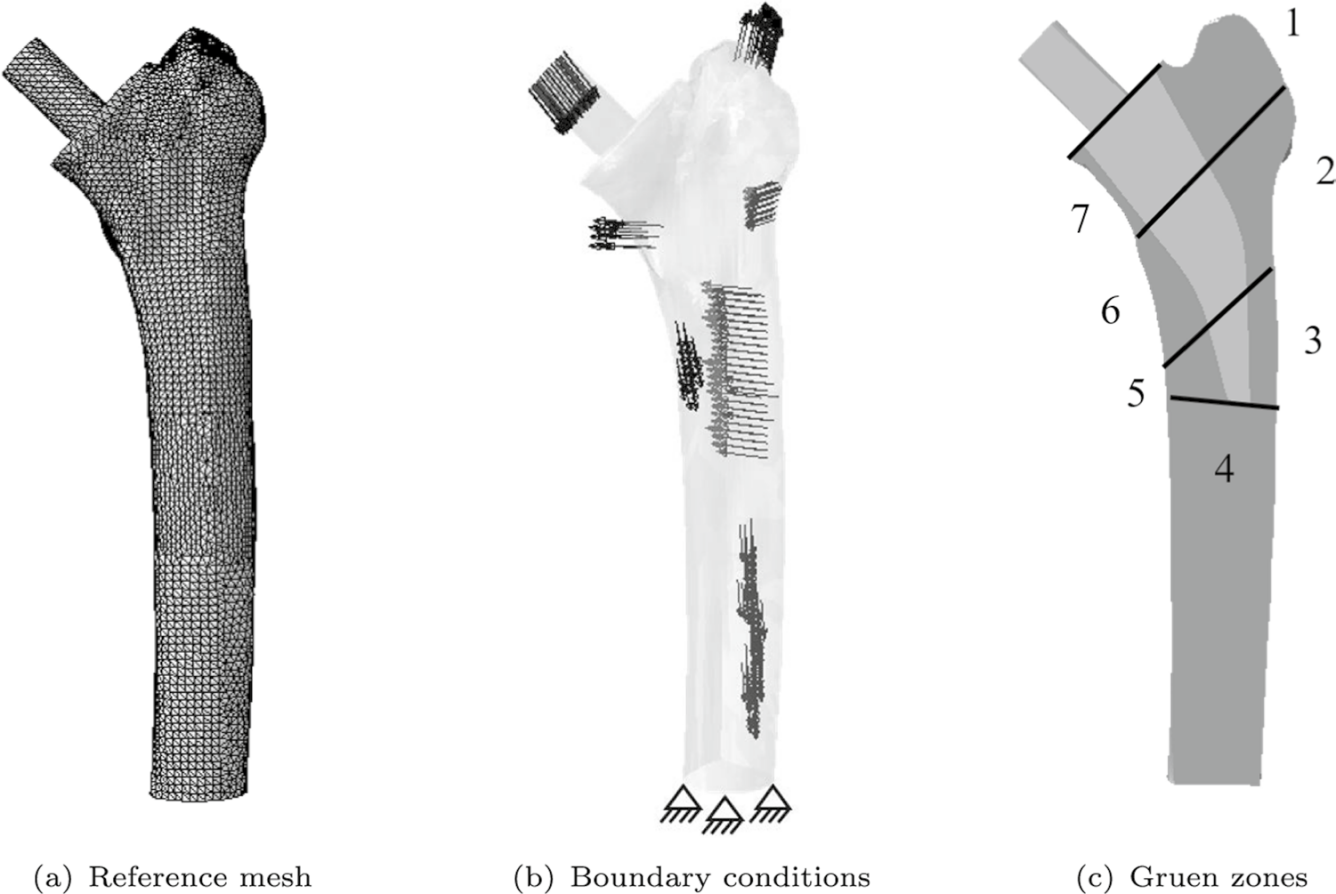
FEM femur model with (a) reference mesh, (b) boundary conditions and (c) the Gruen zones for the reference mesh

In this work, the same human femur geometry with an integrated non-cemented Metha^®^ implant (Aesculap, Tuttlingen, Germany) as described in Lutz (2011) and Bensel et al. (2023) is used. The implantation is modelled as a perfect press-fit. A similar fine mesh size to that used by Bensel et al. (2023) is uniformly applied to accurately represent the geometry. The resulting reference FEM mesh consists of 111041 four-node linear tetrahedral elements with 83296 degrees of freedom and is depicted in Fig. 1(a).

The boundary conditions are adopted from Lutz (2011). These boundary conditions represent a clamping at the bottom and loading by the joint force as well as the six main muscle forces (gluteus maximus, gluteus medius/ minimus, vastus lateralis, illiopsas, biceps femoris and vastus medialis) (see Fig. 1(b)). The loads have been calculated as statically equivalent loads corresponding to the measured BMD distribution projected from CT data to the FEM model (Lutz 2011). For subsequent analysis, the Gruen zones are defined on the reference mesh in Fig. 1(c), according to Gruen et al. (1979).

### Mesh morphing technique

Laplace’s equation is used to morph the nodes of the parameterised reference mesh in Fig. 1(a) to create the mesh for a new implant position. Consequently, the node numbering, total number and connectivity of DOFs are preserved, which facilitates the use of MOR techniques.

The position of the implant is varied according to the parameters $\varvec{\mu } = [\delta x,\delta y, \delta z,\alpha ,\beta ,\gamma ]$, where $\delta _x, \delta _y, \delta _z$ are translations along and $\alpha ,\beta ,\gamma$ rotation angles around the *x*, *y* and *z* axes, respectively. All parameters are varied relative to the reference position ($\delta x = 0~\text {mm},\delta y = 0~\text {mm}, \delta z = 0~\text {mm},\alpha = 0^{\circ },\beta = 0^{\circ },\gamma = 0^{\circ }$). The number of parameters is $n_p = 6$.

**Fig. 2:**
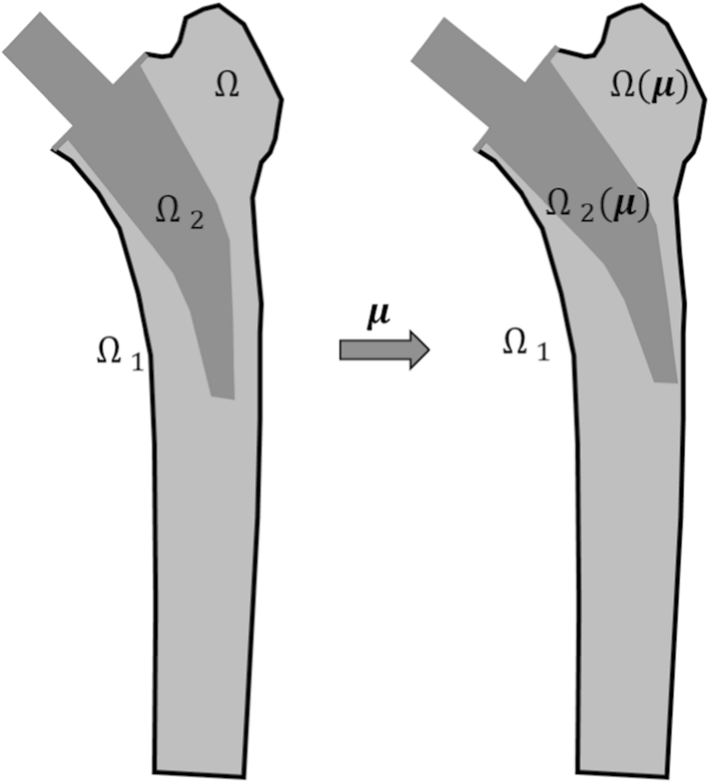
Mesh morphing with Laplace’s equation. $\Omega _1$ are the nodes of the fixed boundary, $\Omega _2$ are the nodes performing a rigid body motion and the remaining nodes on $\Omega$ are calculated using Laplace’s equation

The mesh morphing via Laplace’s equation is defined by

1 $$\begin{aligned} \Delta x_i (\varvec{\mu }) = 0 \quad \text {where} \quad x_i = [x, y, z] \end{aligned}$$

and is subject to the following boundary conditions

2 $$\begin{aligned} {\left\{ \begin{array}{ll} (x,y,z)|_{\Omega _{1}} & = (p,q,r)|_{\Omega _{1}} \\ (x,y,z)|_{\Omega _{2}} & = (g_x (\varvec{\mu }), g_y(\varvec{\mu }), g_z(\varvec{\mu }))|_{\Omega _{2}} \end{array}\right. } . \end{aligned}$$

(*p*, *q*, *r*) are the initial nodal coordinates of the FEM model. $\Omega _1$ contains the nodes of the boundary of the femur. These coordinates remain fixed because a single patient’s femur is considered for this study. $\Omega _2$ contains the nodes of the implant, which is subject to a rigid body motion denoted as $(g_x (\varvec{\mu }), g_y(\varvec{\mu }), g_z(\varvec{\mu }))$. Furthermore, it contains the nodes of the cutting plane to ensure a smooth transition between the implant’s surface and the femur. The boundaries are highlighted in Fig. 2. The new coordinates of the implant nodes performing the rigid body motion can be calculated as

3 $$\begin{aligned} \begin{bmatrix} g_x(\varvec{\mu }) \\ g_y(\varvec{\mu }) \\ g_z(\varvec{\mu }) \\ 1 \end{bmatrix} = \textbf{T}(\varvec{\mu }) \begin{bmatrix} p \\ q \\ r \\ 1 \end{bmatrix}_{\Omega _2} , \end{aligned}$$

where $\textbf{T}(\varvec{\mu })$ is the transformation matrix, defined as

4 $$\begin{aligned}&\textbf{T}(\varvec{\mu }) = \textbf{R}_x \textbf{R}_y \textbf{R}_z \textbf{D} \end{aligned}$$

5 $$\begin{aligned}= \begin{bmatrix} T_{11}(\varvec{\mu }) & T_{12}(\varvec{\mu }) & T_{13}(\varvec{\mu }) & T_{14}(\varvec{\mu }) \\ T_{21}(\varvec{\mu }) & T_{22}(\varvec{\mu }) & T_{23}(\varvec{\mu }) & T_{24}(\varvec{\mu }) \\ T_{31}(\varvec{\mu }) & T_{32}(\varvec{\mu }) & T_{33}(\varvec{\mu }) & T_{34}(\varvec{\mu }) \\ T_{41}(\varvec{\mu }) & T_{42}(\varvec{\mu }) & T_{43}(\varvec{\mu }) & T_{44}(\varvec{\mu }) \end{bmatrix} \begin{matrix} \rightarrow \textbf{T}_x(\varvec{\mu }) \\ \rightarrow \textbf{T}_y(\varvec{\mu }) \\ \rightarrow \textbf{T}_z(\varvec{\mu }) \\ \end{matrix} . \end{aligned}$$

$\textbf{R}_x$, $\textbf{R}_y$ and $\textbf{R}_z$ are the rotation matrices around the respective axis defined as

6a $$\begin{aligned} \textbf{R}_x = \left[ \begin{array}{ccccccc} 1 & & 0 & & 0 & & 0 \\ 0 & & \text {cos} \alpha & & - \text {sin} \alpha & & 0 \\ 0 & & \text {sin} \alpha & & \phantom {-}\text {cos} \alpha & & 0 \\ 0 & & 0 & & 0 & & 1 \\ \end{array}\right] , \end{aligned}$$

6b $$\begin{aligned} \textbf{R}_y = \left[ \begin{array}{ccccccc} \phantom {-}\text {cos} \beta & & 0 & & \text {sin} \beta & & 0 \\ 0 & & 1 & & 0 & & 0 \\ -\text {sin} \beta & & 0 & & \text {cos} \beta & & 0 \\ 0 & & 0 & & 0 & & 1 \\ \end{array}\right] , \end{aligned}$$

6c $$\begin{aligned} \textbf{R}_z = \left[ \begin{array}{ccccccc} \phantom {-}\text {cos} \gamma & & \text {sin} \gamma & & 0 & & 0 \\ - \text {sin} \gamma & & \text {cos} \gamma & & 0 & & 0 \\ 0 & & 0 & & 1 & & 0 \\ 0 & & 0 & & 0 & & 1 \\ \end{array}\right] , \end{aligned}$$

and $\textbf{D}$ is the translation matrix defined as

7 $$\begin{aligned} \textbf{D} = \left[ \begin{array}{ccccccc} 1 & & 0 & & 0 & & \delta x \\ 0 & & 1 & & 0 & & \delta y \\ 0 & & 0 & & 1 & & \delta z \\ 0 & & 0 & & 0 & & 1 \\ \end{array}\right] {.} \end{aligned}$$

Consequently, the new coordinates for the implant nodes, exemplary for the *x*-direction of one node, are calculated as

8 $$\begin{aligned} g_x(\varvec{\mu }) = \textbf{T}_x(\varvec{\mu }) \begin{bmatrix} p \\ q \\ r \\ 1 \end{bmatrix}_{\Omega _2} = \textbf{T}_x (\varvec{\mu }) \textbf{h}_{\Omega _2} . \end{aligned}$$

Laplace’s equation is used to calculate the new coordinates of the remaining nodes. The FEM is used to solve the underlying equations, resulting in the following system of equations

9 $$\begin{aligned} \begin{bmatrix} \textbf{K}_{11} & \textbf{K}_{12} & \textbf{K}_{13} \\ \textbf{K}_{21} & \textbf{K}_{22} & \textbf{K}_{23} \\ \textbf{K}_{31} & \textbf{K}_{32} & \textbf{K}_{33}\\ \end{bmatrix} \begin{bmatrix} \textbf{x}_1(\varvec{\mu }) \\ \textbf{x}_2(\varvec{\mu }) \\ \textbf{x}_3(\varvec{\mu }) \end{bmatrix} = \varvec{0}, \end{aligned}$$

where $\textbf{x}_1(\varvec{\mu }) = \textbf{p}_{\Omega _1}$ are the coordinates of the boundary nodes on $\Omega _1$ with length $n_1$ and $\textbf{x}_2(\varvec{\mu }) = \textbf{G}_x(\varvec{\mu }) = \textbf{H}_{\Omega _2}^T \textbf{T}_x^T(\varvec{\mu })$ the coordinates of the nodes on $\Omega _2$ with length $n_2$ performing the rigid body motion. $\textbf{G}_x(\mathbf {\varvec{\mu }})$ and $\textbf{H}_{\Omega _2}$ are matrices, storing the morphed and initial coordinates of the nodes on $\Omega _2$.

Using static condensation, the unknown $\textbf{x}_3$-coordinates with length $n_3$ of the femur can be calculated as

10 $$\begin{aligned} \textbf{x}_3(\varvec{\mu }) = - \textbf{K}_{33}^{-1} \textbf{K}_{31} \textbf{p}_{\Omega _1} - \textbf{K}_{33}^{-1} \textbf{K}_{32} \textbf{G}_x(\mathbf {\varvec{\mu }}) . \end{aligned}$$

In matrix notation, the coordinates are calculated as follows

11 $$\begin{aligned}&\textbf{x}(\varvec{\mu }) = \begin{bmatrix} \textbf{p}_{\Omega _1} \\ \textbf{H}_{\Omega _2}^T \textbf{T}_x^T(\varvec{\mu }) \\ - \textbf{K}_{33}^{-1} \textbf{K}_{31} \textbf{p}_{\Omega _1} - \textbf{K}_{33}^{-1} \textbf{K}_{32} \textbf{G}_x(\varvec{\mu }) \end{bmatrix} \end{aligned}$$

12 $$\begin{aligned}= \begin{bmatrix} \textbf{p}_{\Omega _1} & \varvec{0} \\ \varvec{0} & \textbf{H}_{\Omega _2}^T \\ - \textbf{K}_{33}^{-1} \textbf{K}_{31} \textbf{p}_{\Omega _1} & - \textbf{K}_{33}^{-1} \textbf{K}_{32} \textbf{H}_{\Omega _2}^T \end{bmatrix} \begin{bmatrix} \textbf{I} \\ \textbf{T}^T_x(\varvec{\mu }) \end{bmatrix}, \end{aligned}$$

where $\textbf{I}$ is the identity matrix. Thus, for the calculation of the new coordinates only $\textbf{T}_x(\varvec{\mu })$ needs to be recalculated. The remaining coordinate directions are treated analogously.

After morphing the parameterised reference mesh, the mesh quality is checked by ensuring that the volumes of every element are not smaller than a certain tolerance threshold.

**Fig. 3:**
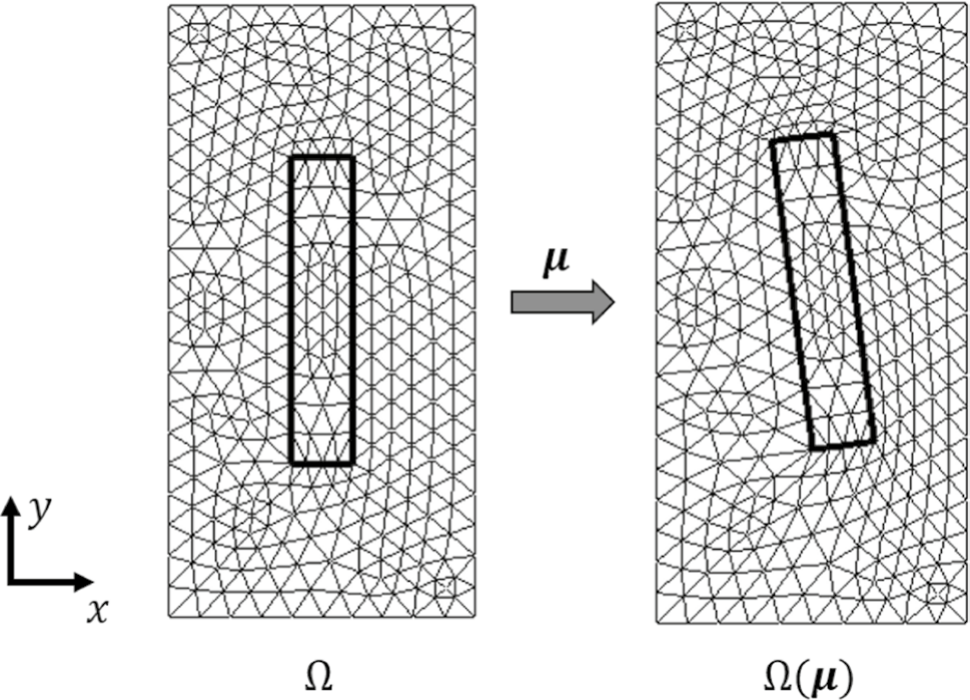
Mesh morphing of example mesh. $\Omega _1$ are the nodes of the fixed outer boundary, $\Omega _2$ are the nodes performing a rigid body motion and the remaining nodes in $\Omega$ are calculated using Laplace’s equation

In Fig. 3, the mesh morphing is applied to an exemplary two-dimensional reference mesh. The boundary nodes on $\Omega _1$ remain fixed, whereas the nodes of the inner rectangle on $\Omega _2$ are the nodes performing a rigid body motion. The mesh of the inner rectangle stays constant, whereas the remaining nodes on $\Omega (\varvec{\mu })$ are morphed by Laplace’s equation. Thus, the node numbering and the total number of DOFs are maintained, which facilitates the usage of model order reduction techniques.

### Bone remodelling material model

For the bone remodelling, the gradient-enhanced bone remodelling material model by Bensel et al. (2023) is applied. All quantities are parameter-dependent on the position of the implant, but the dependence is omitted in the equations for clarity. The model is based on the assumption that the small strain theory is applicable and that the process can be modelled as quasi-static and isothermal.

Further, it is assumed that the free energy $\psi$ and thus the strain energy density $\Psi$ depend on two internal variables: the elastic strain $\varvec{\varepsilon }$ and the BMD $\varrho$. This relationship is expressed as

13 $$\begin{aligned} \Psi ( \varrho , \varvec{\varepsilon } ) = \varrho \psi ( \varrho , \varvec{\varepsilon } ) . \end{aligned}$$

Following the Clausius–Duhem inequality (Nackenhorst 2018), the Cauchy stress $\varvec{\sigma }$ is derived from the strain energy density

14 $$\begin{aligned} \varvec{ \sigma } = \frac{ \partial \Psi }{ \partial \varvec{ \varepsilon } } . \end{aligned}$$

The nonlinear constitutive relation between the BMD and Young’s modulus *E* according to Lutz and Nackenhorst (2010) is used, defined by

15 $$\begin{aligned} E = E_0 \left( \frac{ \varrho }{ \varrho _0 } \right) ^2 , \end{aligned}$$

where $E_0$ and $\varrho _0$ are reference values. This relation is inserted into the generalised Hooke’s law

16 $$\begin{aligned} \varvec{ \sigma } = \mathbb {C} :\varvec{ \varepsilon } , \end{aligned}$$

with the linear elastic material tensor $\mathbb {C}^{\text {LE}}$ derived for a reference state $\varrho _0$, which leads to the constitutive relation

17 $$\begin{aligned} \varvec{ \sigma } = \left( \frac{ \varrho }{ \varrho _0} \right) ^2 \mathbb {C}^{ \text {LE} } :\varvec{ \varepsilon } . \end{aligned}$$

From this relation, the mechanical free energy density $\psi _\text {mech}$ is concluded

18 $$\begin{aligned} \psi _\text {mech}&= \left( \frac{ \varrho }{ \varrho _0} \right) ^2 \psi ^{\text {LE}} \nonumber \\&= \frac{1}{\varrho } \left( \frac{ \varrho }{ \varrho _0} \right) ^2 \left[ \frac{\lambda }{2} \text {tr}( \varvec{\varepsilon })^2 + \mu \text {tr} (\varvec{\varepsilon }^2) \right] , \end{aligned}$$

where $\lambda$ and $\mu$ are the Lamé parameters and $\psi ^{\text {LE}}$ is the linear elastic reference free energy, respectively.

The evolution equation for the BMD, obtained from the balance of mass, is defined as

19 $$\begin{aligned} \dot{\rho } = \frac{\partial \varrho }{\partial t} , \end{aligned}$$

where *t* denotes the process time of the quasi-static simulation. The BMD is limited by physiological limits of the minimum BMD $\varrho _{\text {min}}$ and maximum BMD $\varrho _{\text {max}}$. The mass source $\dot{\rho }$ is defined according to the strain energy density driven bone remodelling formulation by Beaupré et al. (1990) using a first-order approach

20 $$\begin{aligned} \dot{\rho } = c \left( \Psi - \Psi ^\text {ref} \right) = c \left( \varrho \psi - \Psi ^\text {ref} \right) . \end{aligned}$$

Here, *c* is a model parameter that describes the speed of the remodelling process and $\Psi ^\text {ref}$ is a physiological target value.

The system of partial differential equations can be summarised as

21 $$\begin{aligned} {\left\{ \begin{array}{ll} \nabla \cdot \varvec{\sigma }\left( \varvec{\varepsilon }, \varrho \right) = 0 \quad & \text {in } \Omega \times \mathbb {T} \\ \hspace{0.5cm} \text {with } \varvec{\varepsilon } = \nabla ^\text {s}\textbf{u} \\ \hspace{0.5cm} \text {and } \varvec{\sigma } = \mathbb {C}\left( \varrho \right) :\varvec{\varepsilon } \\ \dot{\rho } = c \left( \Psi - \Psi ^\text {ref} \right) \quad & \text {in } \Omega \times \mathbb {T} \end{array}\right. } . \end{aligned}$$

The equations denote the mechanical equilibrium of forces under the omission of body forces and the balance of mass without mass fluxes, respectively. $\nabla ^\text {s}$ denotes the symmetric gradient, $\Omega$ the spatial domain and $\mathbb {T}$ the temporal or process time domain.

The corresponding boundary and initial conditions are defined as

22 $$\begin{aligned} {\left\{ \begin{array}{ll} \textbf{u} = \textbf{u}_\text {D} \quad & \text {in } \partial \Omega _\text {D} \times \mathbb {T} \\ \textbf{n} \cdot \varvec{\sigma } = \textbf{t} \quad & \text {in } \partial \Omega _\text {N} \times \mathbb {T} \\ \varrho ^{(0)} = \varrho _\text {init} \quad & \text {in } \Omega \times 0 \end{array}\right. } . \end{aligned}$$

$\partial \Omega _D$ denotes the Dirichlet boundary and $\partial \Omega _N$ denotes the Neumann boundary. $\textbf{t}$ is the surface traction, $\textbf{n}$ is the normal direction and $\varrho _\text {init}$ is the initial BMD, respectively.

The FEM is used to solve the nonlinear system of equations

23 $$\begin{aligned} \textbf{K} (\textbf{u}) \Delta \textbf{u} = \textbf{f} , \end{aligned}$$

where $\textbf{K}$ is the tangent stiffness matrix dependent on the displacements $\textbf{u}$ and $\textbf{f}$ is the non-equilibrium force vector. The Newton–Raphson method is used to solve the system of equations.

To avoid the so-called checkerboarding phenomenon (Bensel et al. 2023), a gradient enhancement approach is applied to the free energy

24 $$\begin{aligned} \Psi (\varvec{\varepsilon }, \varrho , \phi ) = \varrho \psi&= \varrho \psi _{\text {mech}}(\varvec{\varepsilon }, \varrho ) + \frac{ \alpha _{\text {GE}} }{2} (\phi - \varrho )^2 \nonumber \\&+ \frac{\beta _{\text {GE}}}{2} \mid \nabla \phi \mid ^2 , \end{aligned}$$

where $\alpha _{\text {GE}}$ and $\beta _{\text {GE}}$ are model parameters, and $\phi$ is the nodal representation of the BMD field. $\alpha _{\text {GE}}$ controls the coupling of $\phi$ and $\varrho$ whereas $\beta _{\text {GE}}$ controls the penalisation of the gradient of $\phi$.

Further, an implicit Euler scheme is used to perform the internal variable update to compute the local BMD field (Bensel et al. 2023; Bittens and Nackenhorst 2023)

25 $$\begin{aligned} \varrho ^{(i+1)}&= \varrho ^{(i)} + \Delta \varrho \nonumber \\&= \varrho ^{(i)} + \Delta t \cdot \dot{\rho }( \varvec{\varepsilon }^{(i+1)}, \varvec{\phi }^{(i+1)}) . \end{aligned}$$

For a detailed explanation of the numerical treatment, it is referred to our previous work (Bensel et al. 2023).

For the numerical solution, the commercial FEM software Abaqus (Abaqus 2017, Dassault Systèmes, Vélizy-Villacoublay, France) is used. The phenomenological constitutive bone remodelling model described above is employed for the femur using a user element (UEL) subroutine (Bensel and Reiber 2022). The corresponding parameter values are summarised in Table 1. To establish the initial BMD distribution of the femur, the biomechanically equilibrated BMD distribution for the complete femur (with head) is projected onto the model with the implant. For the implant, a linear elastic material of titanium with a Young’s modulus $E = 105000$ $\text {N/mm}^2$ and a Poisson’s ratio $\nu = 0.3$ is used.

**Table 1:** Bone remodelling simulation parameters

**Parameter**	**Value**	**Unit**
$E_0$	6500.0	[$\text {N}/\text {mm}^2$]
$\nu$	0.3	[-]
$\varrho _0$	1.0	[$\text {g}/\text {cm}^3$]
$\Psi _\text {ref}$	0.002	[$\text {N}/\text {mm}^2$]
*c*	0.01	[$\text {s}/\text {m}^2$]
$\varrho _\text {min}$	0.001	[$\text {g}/\text {cm}^3$]
$\varrho _\text {max}$	2.0	[$\text {g}/\text {cm}^3$]
$\alpha _\text {GE}$	0.01	[$\text {m}^5/\text {s}^2\text {kg}$]
$\beta _\text {GE}$	$10^{-8}$	[$\text {m}^7/\text {s}^2\text {kg}$]

**Fig. 4:**
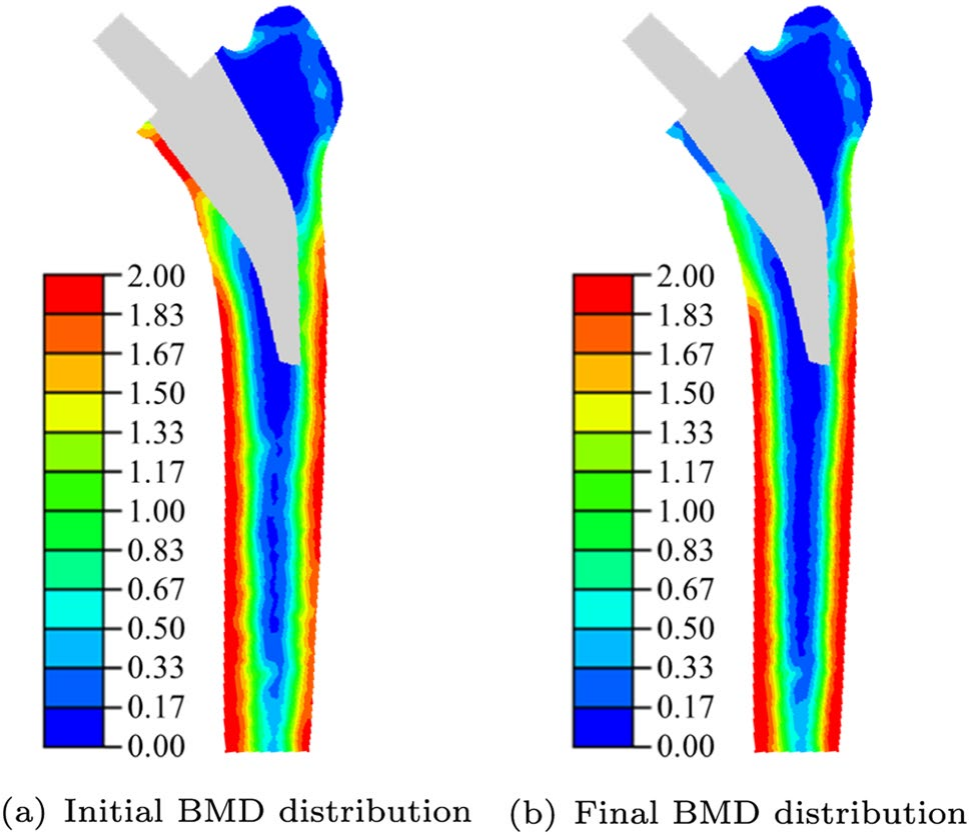
Initial (a) and final (b) BMD distribution ($\phi$ in $\text {g}/\text {cm}^3$) for reference position of hip implant ($\delta _x = 0$ mm, $\delta _y = 0$ mm, $\delta _z = 0$ mm, $\alpha = {0}^\circ$, $\beta = {0}^\circ$ and $\gamma = {0}^\circ$)

In the first simulation step, the loads are applied, and during the subsequent step, several quasi-static iterations are performed until the BMD distribution converges. The initial and the final BMD distribution for the reference position are depicted in Fig. 4. The corresponding mass changes in per cent in the Gruen zones are given in Table 2. A comparison of these results with the simulation results obtained by Lutz (2011) can be performed (see Table 2). The simulation results exhibit similar trends. The most substantial mass increase is observed in Gruen zone 1, while the largest decreases occur in Gruen zones 6 and 7. Present differences can be attributed to the variations in the reference implant position tested and the different definitions of the Gruen zones. The mass decrease in Gruen zone 7 can be attributed to the phenomenon of stress shielding. The higher stress shielding effect observed in the simulations can be attributed to the ideal press-fit conditions used (Lutz 2011). For a direct comparison with clinical data, a clinical study providing the information to precisely define the initial and boundary conditions for the numerical simulation is required.

**Table 2:** Mass change in % in Gruen zones for the reference position of the current work and for the numerical results by Lutz (2011)

**Gruen zone**	Current work	Lutz (2011)
1	10.2	19.1
2	$-$7.4	$-$8.8
3	$-$7.0	$-$4.3
4	0.6	$-$1.9
5	$-$1.0	$-$10.0
6	$-$29.1	$-$35.4
7	$-$61.9	$-$87.8

## The reduced-order model

**Fig. 5:**
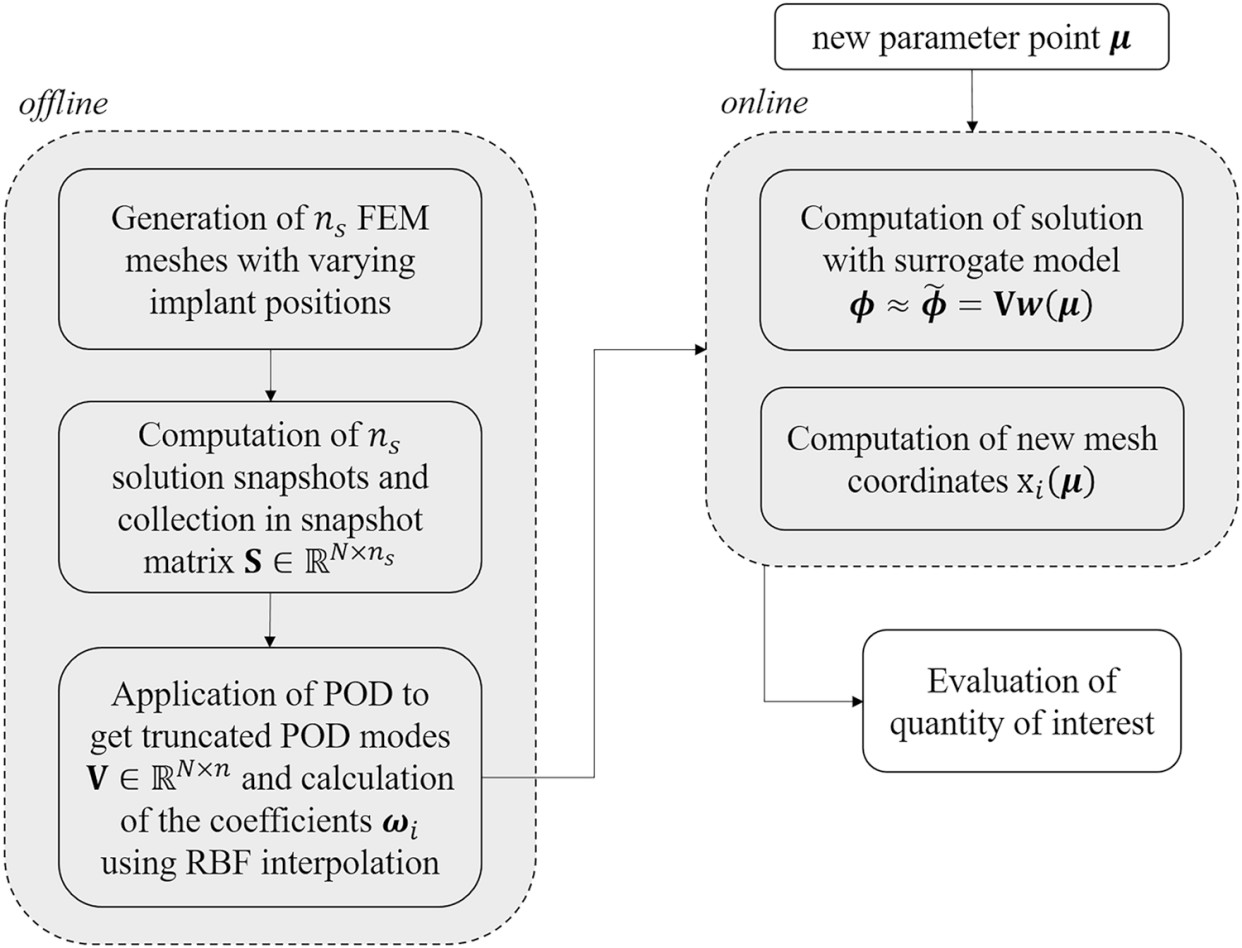
Set-up of surrogate model with offline and online stage

The set-up of the reduced-order model is divided into an offline and an online phase, as sketched in Fig. 5. In the offline phase, several computationally expensive high-fidelity bone remodelling simulations for different implant positions are performed, as described in Sect. 2.

Using these high-fidelity solution snapshots, a surrogate model is set up so that the full BMD solution $\varvec{\phi }$ for a new set of parameters $\varvec{\mu }$ for the implant position can be approximated as a linear combination of reduced basis functions $\textbf{V}$ and the corresponding weights $\varvec{w}(\varvec{\mu })$

26 $$\begin{aligned} \varvec{\phi } \approx \tilde{\varvec{\phi }} = \varvec{V} \varvec{w}(\varvec{\mu }) . \end{aligned}$$

In this work, the reduced basis $\textbf{V}$ are the truncated POD modes (see Sect. 3.1), and the corresponding weights $\varvec{w}(\varvec{\mu })$ are calculated using RBF interpolation (see Sect. 3.2). The POD modes reflect the spatial variation of the BMD distribution and the RBFs incorporate the parameter dependency, which, in this case, is the changing implant position.

### Proper orthogonal decomposition

To compute the POD modes, $n_s$ high-fidelity simulations at different parameter points are computed. The BMD distribution $\varvec{\phi }_i=\varvec{\phi }(\varvec{\mu }_i)$ denotes the $i^{\text {th}}$ solution, where $\varvec{\mu }_i$ is the corresponding parameter vector. The solutions are called snapshots and are collected in the corresponding snapshot matrix $\textbf{S} = [ \varvec{\phi }_1, \cdots , \varvec{\phi }_{n_s} ] \in \mathbb {R}^{N \times n_s}$, where *N* is the number of nodes and $n_s$ is the number of snapshots. The corresponding parameter vectors are stored in the parameter matrix $\textbf{P} = [ \varvec{\mu }_1, \cdots , \varvec{\mu }_{n_s} ]$.

Next, using singular value decomposition (SVD), a matrix decomposition of $\textbf{S}$ is performed

27 $$\begin{aligned} \textbf{S} = \textbf{U} \mathbf {\Sigma } \textbf{Z}^T ,\end{aligned}$$

where $\mathbf{U} = [ \varvec{\xi }_1, \cdots , \varvec{\xi }_{N} ] \in \mathbb {R}^{N \times N}$ and $\mathbf{Z} = [ \varvec{\zeta }_1, \cdots , \varvec{\zeta }_{n_s} ] \in \mathbb {R}^{n_s \times n_s}$ are orthogonal matrices containing the left and right singular vectors and $\mathbf {\Sigma } = \text {diag}(\varsigma _1, \cdots , \varsigma _{n_s}) \in \mathbb {R}^{N \times n_s}$ is a matrix containing the singular values of $\textbf{S}$, where $\varsigma _1 \ge \cdots \ge \varsigma _{n_s} \ge 0$.

The POD modes $\textbf{V} = [ \varvec{\xi }_1, \cdots , \varvec{\xi }_{n} ]$ are a subset of $\textbf{U}$ with the *n* left singular vectors of $\textbf{U}$ corresponding to the *n* largest singular values. The POD modes are orthonormal basis functions and independent of the parameters $\varvec{\mu }$ (Quarteroni et al. 2015).

To choose the number of POD modes *n*, the relative "energy", captured by the POD modes, is chosen to be larger than some tolerance $\kappa$ (Chinesta et al. 2017), which is often 99,9% or more

28 $$\begin{aligned} \frac{\sum ^n_{i=1} \varsigma ^2_i}{\sum ^{n_s}_{i=1} \varsigma ^2_i} > \kappa . \end{aligned}$$

The error in snapshot representation is given by

29 $$\begin{aligned} \epsilon (n) = \sqrt{\frac{\sum ^{n_s}_{i=n} \varsigma ^2_i}{\sum ^n_{i=n_s} \varsigma ^2_i}} . \end{aligned}$$

A reduced snapshot matrix can be calculated as

30 $$\begin{aligned} \textbf{S}_r = \textbf{V}^T \textbf{S} , \end{aligned}$$

from which the original snapshot matrix could be approximated as

31 $$\begin{aligned} \textbf{S} \approx \textbf{V} \textbf{S}_r . \end{aligned}$$

### Radial basis function interpolation

RBFs have been invented for scattered data interpolation (Hardy 1971; Biancolini 2017). This RBF interpolation can be used to calculate the weights in Equation (26) as

32 $$\begin{aligned} \varvec{w}(\varvec{\mu }) = \sum ^{n_s}_{i=1} \varvec{\omega }_i \varphi (\Vert \varvec{\mu } - \varvec{\mu }_i \Vert ) = \sum ^{n_s}_{i=1} \varvec{\omega }_i \varphi ( r ) , \end{aligned}$$

where $\varvec{\omega }_i$ are the RBF coefficients to be determined and $\varphi (\cdot )$ is the RBF. $\Vert \cdot \Vert$ denotes the Euclidean norm. Thus, $\Vert \varvec{\mu } - \varvec{\mu }_i \Vert$ can be regarded as the "radial" distance *r* between a new parameter vector $\varvec{\mu }$ and a reference parameter vector $\varvec{\mu }_i$ from the parameter matrix. All parameter inputs are normalised to eliminate the influence of different scales.

Following Equation (30) and Equation (32), the coefficients $\varvec{\omega }_i$ are determined by ensuring alignment with the given data, which results in the linear system

33 $$\begin{aligned} \textbf{S}_r = \textbf{A} \textbf{B} , \end{aligned}$$

where $\textbf{A} \in \mathbb {R}^{n \times n_s}$ contains the coefficient vectors $\textbf{A} = [\varvec{\omega }_1, \cdots , \varvec{\omega }_{n_s}]$ and $\textbf{B} \in \mathbb {R}^{n_s \times n_s}$ is the interpolation matrix evaluating the RBFs at all parameter combinations $\textbf{B}= [\text {B}_{ij}]= [\varphi (\Vert \varvec{\mu }_i - \varvec{\mu }_j \Vert )]$ with $i,j = 1, \dots , n_s$.

Thus, the coefficients can be determined by solving

34 $$\begin{aligned} \textbf{A} = \textbf{S}_r \textbf{B}^{-1} . \end{aligned}$$

Different kernel functions can be used as an RBF. To guarantee a unique solution, the $\textbf{B}$ matrix must be positive definite. If the kernel is strictly positive definite, a unique solution is available. Otherwise, a polynomial term $h(\varvec{\mu })$ needs to be added to get a well-posed problem, such that

35 $$\begin{aligned} \varvec{w}(\varvec{\mu }) = \sum ^{n_s}_{i=1} \varvec{\omega }_i \varphi (\Vert \varvec{\mu } - \varvec{\mu }_i \Vert ) + h(\varvec{\mu }) , \end{aligned}$$

where

36 $$\begin{aligned} h(\varvec{\mu }) = \sum ^{n_p+1}_{j=1} \varvec{\eta }_j p_j(\varvec{\mu }) . \end{aligned}$$

Consequently, $n_p+1$ additional conditions

37 $$\begin{aligned} \sum ^{n_s}_{i=1} \varvec{\omega }_i p_j(\varvec{\mu }) \overset{!}{=}\ \varvec{0} \quad j = 1, \dots , n_p + 1 \end{aligned}$$

need to be satisfied, which leads to the system

38 $$\begin{aligned} \begin{bmatrix} \textbf{B}^{\phantom {T}} & \textbf{P} \\ \textbf{P}^T & \textbf{0} \end{bmatrix} \begin{bmatrix} \textbf{A} \\ \varvec{\eta } \end{bmatrix} = \begin{bmatrix} \textbf{S}_r\\ \varvec{0} \end{bmatrix} . \end{aligned}$$

Exemplary RBFs are defined in Table 3 where *a* is a shape parameter and *r* is the radial distance. A smaller value for the shape parameter corresponds to a flatter basis function. The Gaussian (G), Inverse multiquadratic (IMQ) and Matérn RBFs (MC0, MC2, MC4) lead to a well-posed system, whereas the multiquadratic (MQ) RBF is only conditionally positive definite (Schaback 2007).

Various methods exist to select the optimal shape parameter of the RBF. A standard strategy is to plot the approximation error for various shape parameters and select the parameter that minimises this error. Other methods include a leave-one-out cross-validation, a maximum likelihood estimator or an effective condition number (Chen et al. 2023; Rippa 1999; Fasshauer and Zhang 2007; Mongillo 2011).

**Table 3:** Radial basis functions where *r* is the Euclidean distance between two parameter points and *a* is a user-defined shape parameter

**RBF**	$\varphi (r)$
Gaussian (G)	$e^{- (a \cdot r)^2}$
Multiquadratic (MQ)	$\sqrt{1+(a \cdot r)^2}$
Inverse multiquadratic (IMQ)	$\frac{1}{\sqrt{1+(a \cdot r)^2}}$
Matérn $C^0$ (MC0)	$e^{-a \cdot r}$
Matérn $C^2$ (MC2)	$e^{-a \cdot r} (1+ a r)$
Matérn $C^4$ (MC4)	$e^{-a \cdot r} (3 +3 a r + (a r)^2)$

## Numerical results

First, the generation of the snapshot data is described (see Sect. 4.1). Next, these snapshot data are analysed to determine whether some parameters could be excluded from the set-up and the analysis of the surrogate model (see Sect. 4.2). Finally, the accuracy and time consumption of the surrogate model are compared to the high-fidelity model in Sect. 4.3.

### High-fidelity simulations

For the generation of the snapshot matrix, an equidistant sampling with three points in every dimension in the range of $\delta x,\delta y, \delta z \in [-1, 1]~\text {mm}$ and $\alpha , \beta , \gamma \in [-1,1]^\circ$ is performed. In total, 729 snapshots are created. For some snapshots, inverted elements are present at the cutting plane of the femur. As a quick remedy, the order of the nodes for these elements is changed without affecting the position of the nodes and the overall node numbering.

Further, the initial BMD of the femur without the implant needs to be projected to the morphed reference mesh because the position of the implant changes. Consequently, the bone remodelling simulation is performed on all 729 morphed reference meshes and the final result of $\varvec{\phi }$ combined with the parameter combination $\varvec{\mu }$ is stored in the snapshot data. The joint load is assumed to remain the same for different implant positions. Due to mesh morphing, the load is transferred directly to the morphed reference mesh without needing pre-processing.

For the computations, a workstation Intel(R) Core(TM) i7-6700 CPU @ 3.40GHz with 32GB RAM was used. The time for the overall mesh generation in the offline phase is 6.3 minutes. Each bone remodelling simulation takes about 25 minutes.

### Sensitivity analysis

The snapshot data are analysed to determine whether some parameters could be excluded from the set-up of the surrogate model. Further, the data are used for the analysis of the surrogate model. The total mass change is examined, and a sensitivity analysis is performed.

**Fig. 6:**
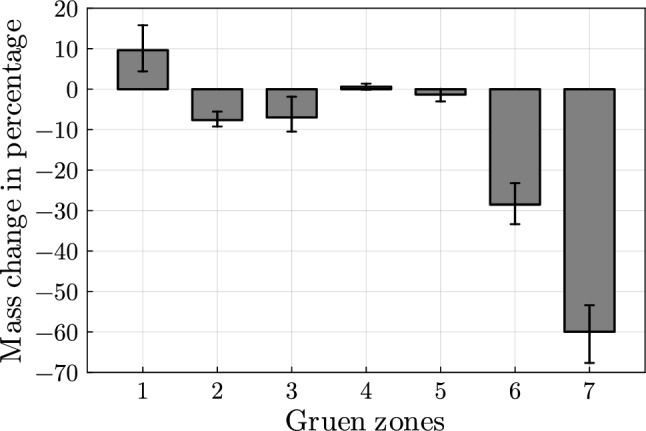
Median mass change with maximum and minimum deviation in Gruen zones for all snapshots

In Fig. 6, the median mass change in percentage for all snapshots is depicted. For this calculation, the BMD at the centre of the elements is multiplied by the element volume. The results conform well with the numerical study from Lutz (2011). Similar to the results for the reference position (see Table 2), the mass decreases most in Gruen zone 7 with a median of 60%. The mass decrease can be explained by the phenomenon of stress shielding.

A global sensitivity analysis is performed using "Spearman’s rank correlation coefficient", which considers nonlinear dependencies based on the rank of the sample output. It is defined as

39 $$\begin{aligned} \rho = \frac{\text {cov} (R(Y), R(X)) }{\sigma _{R(X)} \sigma _{R(Y)}} , \end{aligned}$$

where $\text {cov} (R(Y), R(X))$ is the covariance of the rank variables and $\sigma _{R(X)}$ and $\sigma _{R(Y)}$ are the standard deviations of the rank variables.

**Fig. 7:**
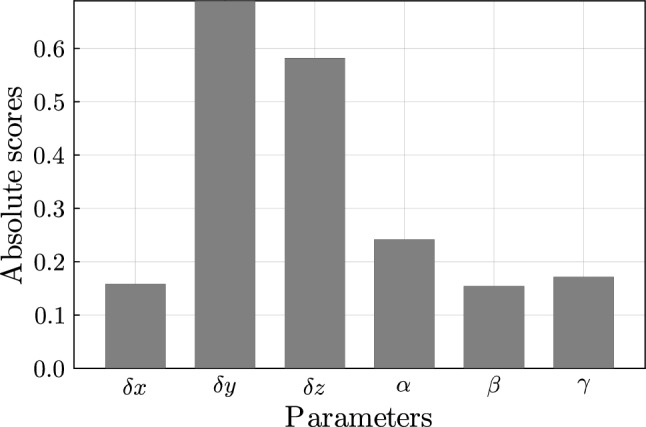
Cross correlation for parameters of surrogate model

**Fig. 8:**
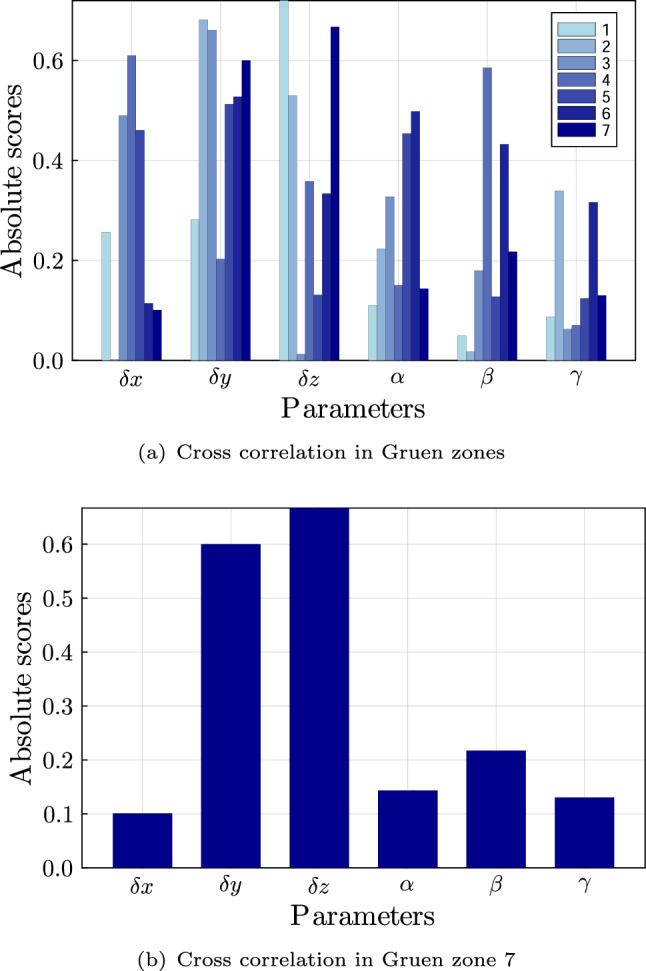
Cross-correlation in all Gruen zones (a) and in Gruen zone 7 (b)

In Fig. 7, Spearman’s rank correlation coefficient is shown for all parameters. The highest influence is present for the displacement in the y-direction and z-direction. Lower influences are found for the other parameters. Nevertheless, upon examining the influence in the different Gruen zones (see Fig. 8(a)), it is evident that the x displacement is important in certain zones, e.g. Gruen zones 4 and 1. It is concluded that no parameter can be excluded from the surrogate model, as each parameter influences the model to some extent. In Gruen zone 7, where the average mass decrease is the greatest (see Fig. 6), the displacements in y- and z-directions have the greatest influence (see Fig. 8(b)). However, all parameters are retained to set up the surrogate model to ensure a comprehensive representation of changes across all Gruen zones.

### Reduced-order model

The time to set up the surrogate model, including the calculation of the POD modes and the RBF coefficients, takes approximately 2.7 seconds and is performed only once. Calculating a new parameter point only takes about 1.2 milliseconds compared to 25 minutes for the high-fidelity simulation. The corresponding calculation of the parameterised mesh, according to Equation (12), takes 0.38 seconds because the matrices can be stored and only $\textbf{T}(\varvec{\mu })$ needs to be recalculated.

**Fig. 9:**
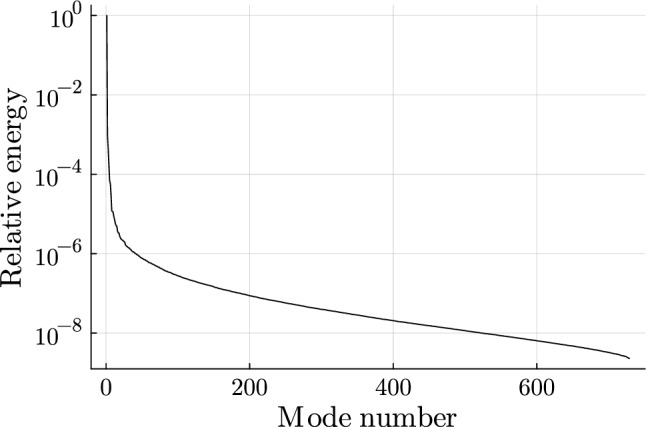
Energy decay of POD modes

**Fig. 10:**
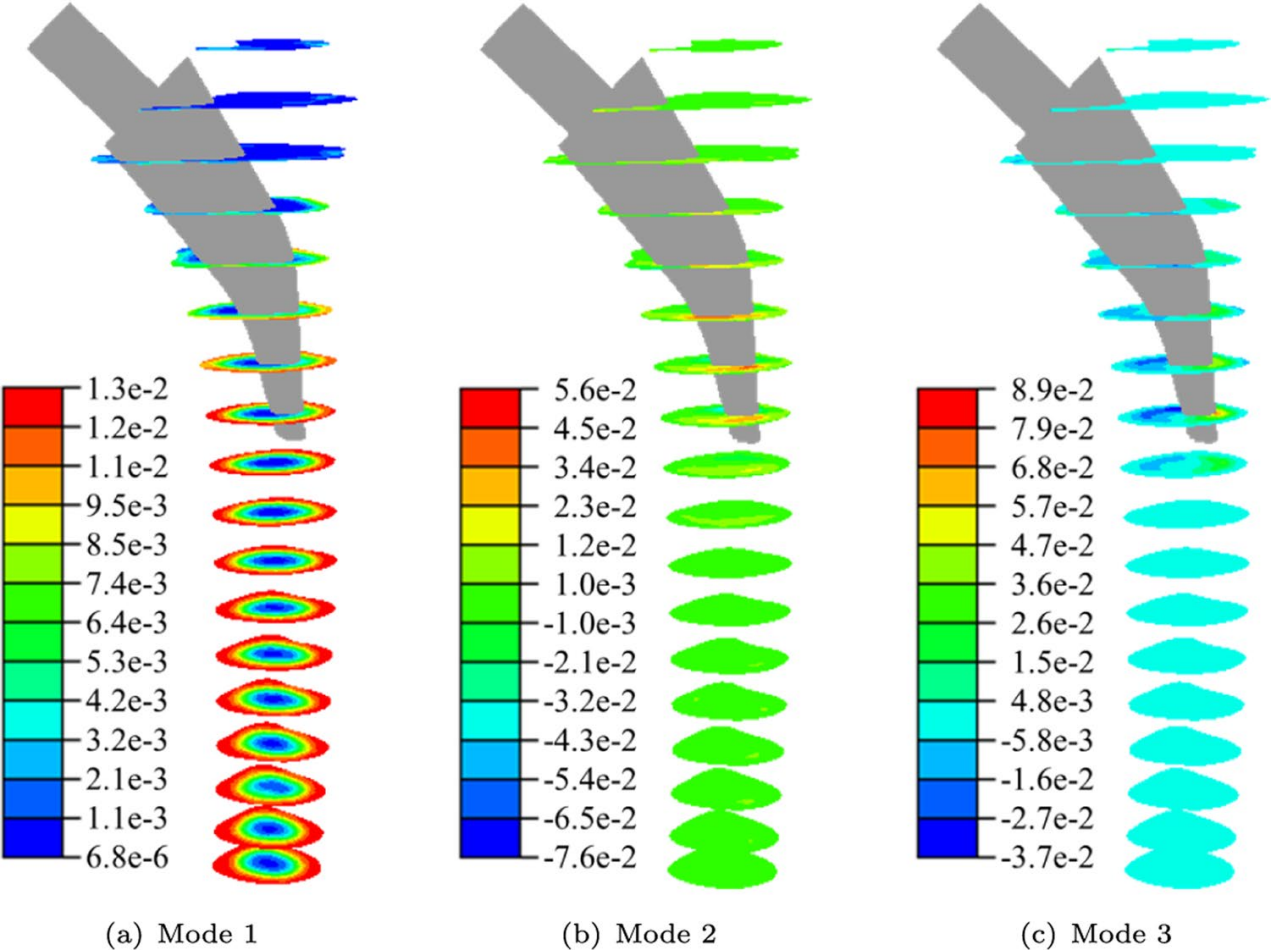
Normalised POD modes 1, 2 and 3

For the set-up of the surrogate model, 17 POD modes are selected as the reduced basis. Thus, a total energy of $99.99\%$ is captured with a POD error of $1.01\%$ (see Fig. 9). The three largest normalised POD modes are depicted in Fig. 10. POD mode 1 captures most of the tubular bone structure. POD modes 2 and 3 capture anterior/posterior and lateral/medial variations around the implant, respectively.

**Fig. 11:**
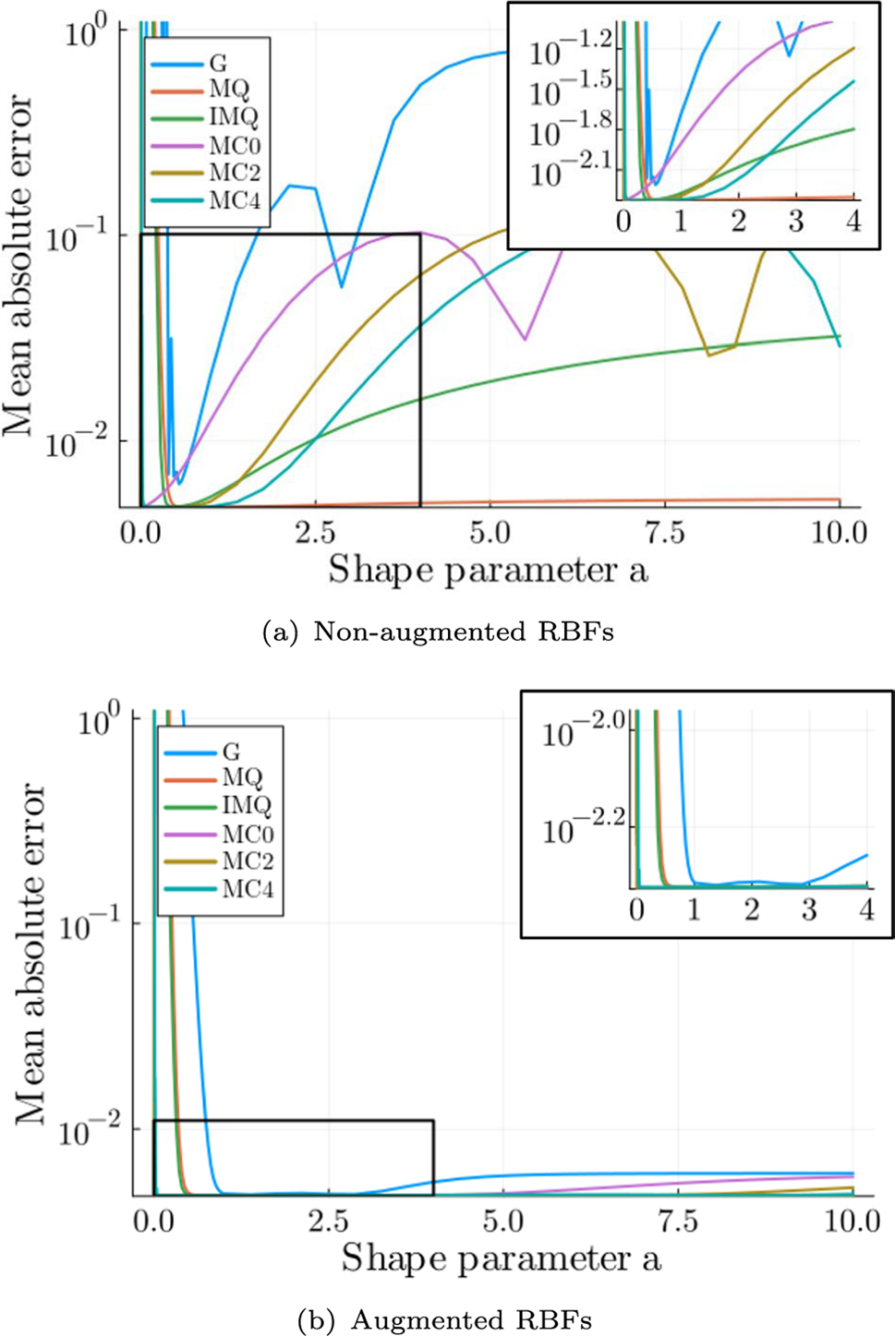
Mean absolute error for different shape parameters for selected non-augmented (a) and augmented (b) RBFs

A test set with 20 pseudorandom samples in the parameter space is calculated and used to analyse the accuracy of the surrogate model. The mean absolute error (MAE) *e* between the surrogate solution and the high-fidelity solution is calculated as

40 $$\begin{aligned} e_\text {abs} = \frac{1}{n_\textrm{dof}}\sum ^{n_\textrm{dof}}_{i=1} |\varvec{\phi }_i - \tilde{\varvec{\phi }}_i | , \end{aligned}$$

where $n_\textrm{dof}$ is the number of nodes of the femur. The mean of the MAE is taken for all 20 parameter combinations. The MAE for different shape parameters for non-augmented and augmented RBFs from Table 3 are plotted in Fig. 11.

For both non-augmented and augmented RBFs, there is a trade-off between high errors at low values and increasing errors for larger shape parameters. For the augmented RBFs, a decrease in the error for larger shape parameters is present. The trade-off is present because for larger shape parameters the RBFs are narrower and there is no reproduction quality between the spikes. In contrast, when the shape parameter approaches zero, the RBFs become wider and the system becomes badly conditioned, causing instability (Schaback 2007). For the non-augmented RBFs, the increasing error at low shape parameters is present for all RBFs except the MC0 RBF, and the increase at larger shape parameters is present for all RBFs except the MQ RBF (see Fig. 11(a)). For the augmented RBFs, the errors decrease compared to the non-augmented RBFs (see Fig. 11(b)). However, the minima of all augmented RBFs are similar and close to 0.0047.

For the latter analysis, the augmented RBF MC0 with a shape parameter of $a=0.0001$ with an MAE of 0.00470 is chosen because the RBF depicts a consistently low error in the chosen parameter range. The MAE in the different Gruen zones is depicted in Table 4. The MAE is the smallest in Gruen zone 4, with a value of 0.0006. In Gruen zone 4, the mass change is the smallest, so changes in the BMD distribution are small between different parameter combinations and are easily represented by the chosen POD modes. In contrast, in Gruen zone 7, the largest error with 0.021 is present. In Gruen zone 7, the largest mass change is present, which needs to be captured by the POD modes.

**Table 4:** Mean absolute error in different Gruen zones for POD-RBF surrogate model (augmented RBF MC0 with $a=0.0001$)

Gruen zone	MAE
1	0.0050
2	0.0045
3	0.0087
4	0.0006
5	0.0047
6	0.0082
7	0.0214

**Fig. 12:**
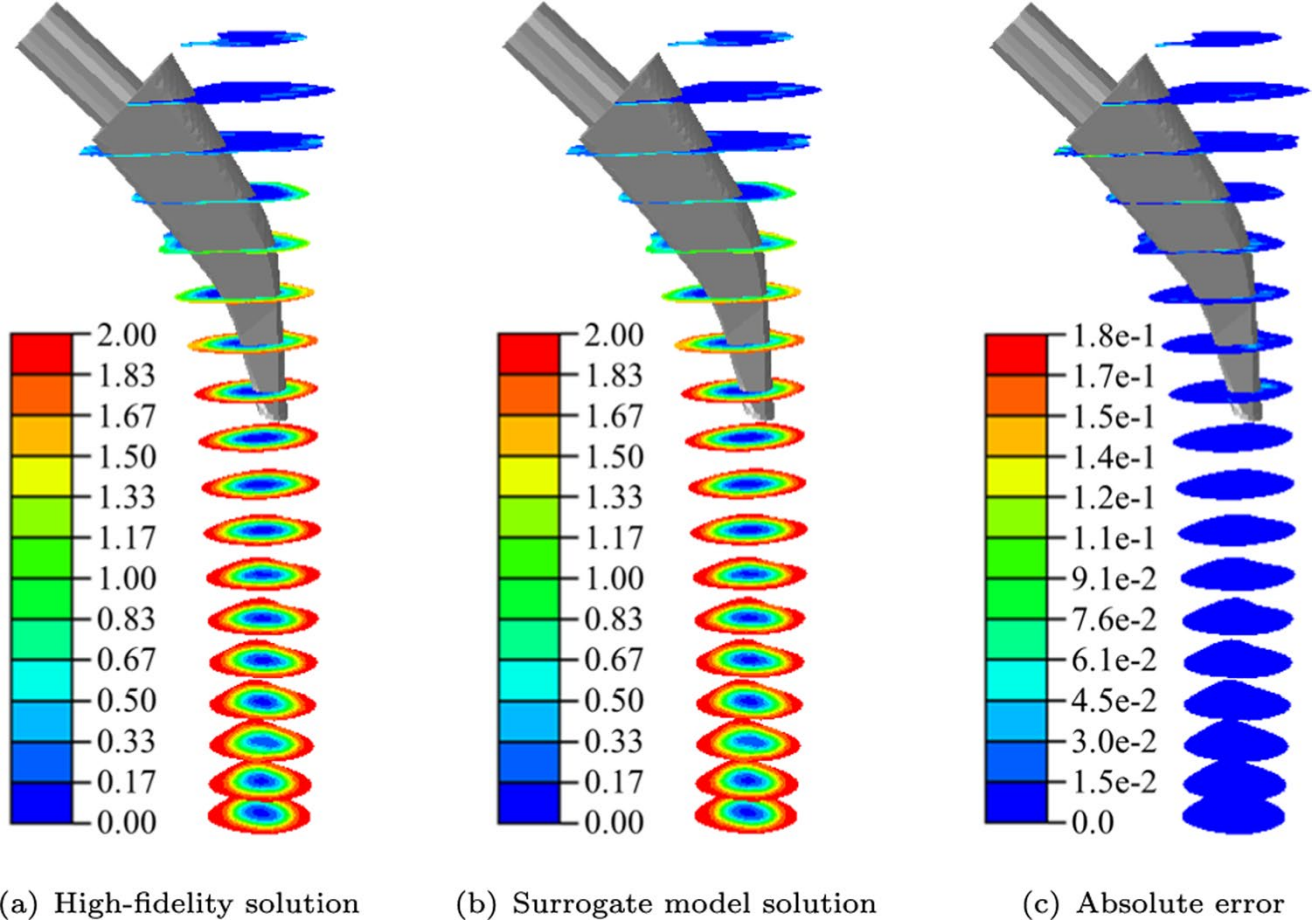
Results for final BMD $\phi$ distribution in $\text {g}/\text {cm}^3$ for a pseudorandom parameter point ($\delta _x = 0.30$ mm, $\delta _y = -0.59$ mm, $\delta _z = 0.59$ mm, $\alpha = {-0.34}^\circ$, $\beta = {0.29}^\circ$ and $\gamma = {0.87}^\circ$) (a) high-fidelity solution (b) surrogate model solution (c) absolute error

**Fig. 13:**
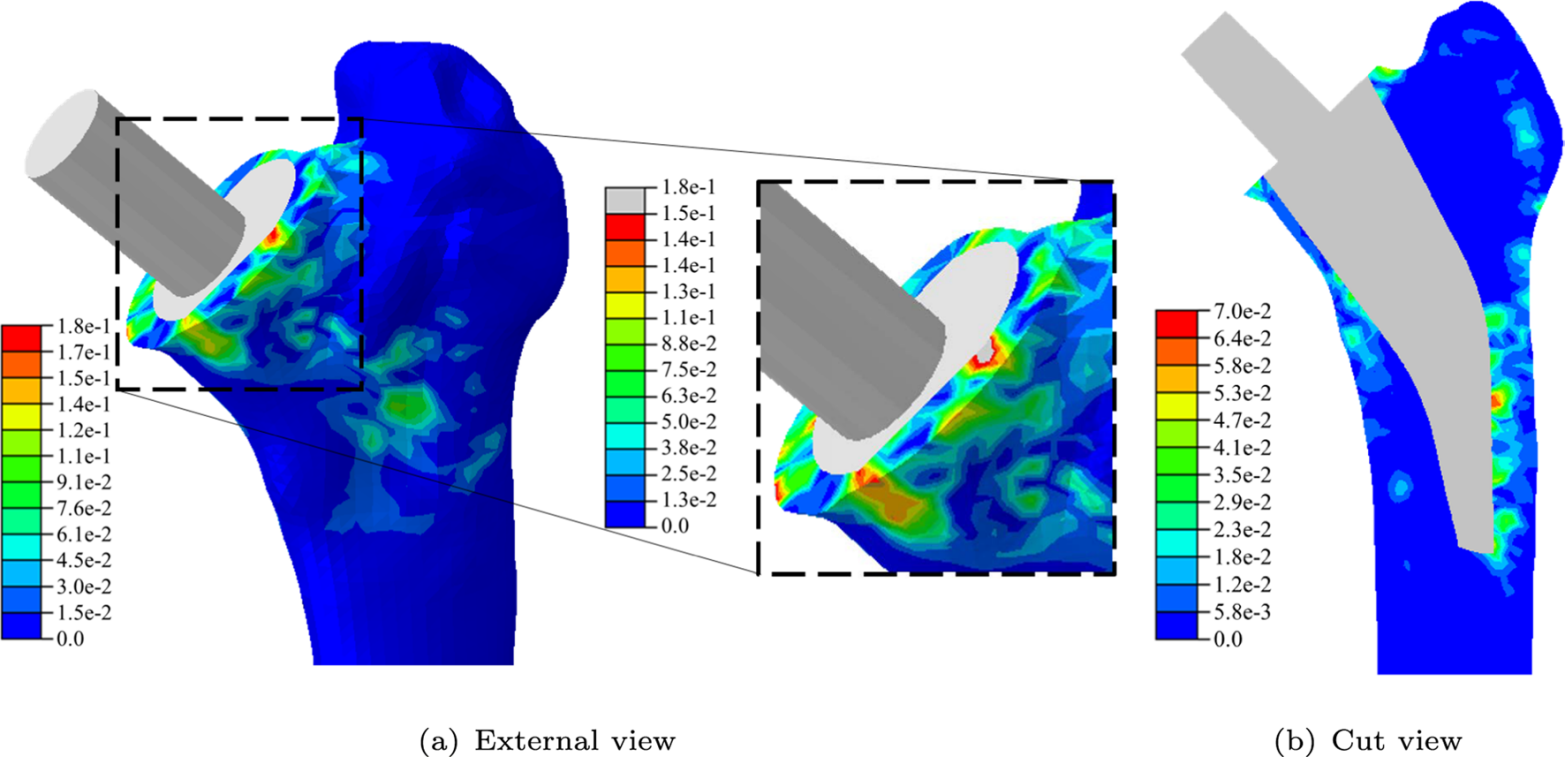
Absolute error of high-fidelity model compared to surrogate model for final BMD distribution ($\phi$ in $\text {g}/\text {cm}^3$) for a pseudorandom parameter point ($\delta _x = 0.30$ mm, $\delta _y = -0.59$ mm, $\delta _z = 0.59$ mm, $\alpha = {-0.34}^\circ$, $\beta = {0.29}^\circ$ and $\gamma = {0.87}^\circ$)

In Fig. 12, the results for the high-fidelity solution, the surrogate model solution and the absolute error for one exemplary position of the random samples ($\delta _x = 0.30$ mm, $\delta _y = -0.59$ mm, $\delta _z = 0.59$ mm, $\alpha = {-0.34}^\circ$, $\beta = {0.29}^\circ$ and $\gamma = {0.87}^\circ$) are shown. Qualitatively, the BMD distributions of the high-fidelity model and the surrogate model coincide. Since the surrogate model provides the solution on the reference mesh, post-processing is required to create the corresponding mesh for visualisation (see Fig. 5). In Fig. 13, the absolute errors are shown in more detail. Examining the absolute errors on the surface in Fig. 13(a), a maximum error of 0.18 is present, which appears at the cutting plane. The maximum error is localised and smaller values occur in the surroundings. For the cut view in Fig. 13(b), smaller errors are present with a localised maximum of 0.07.

## Discussion

Using the POD-RBF method makes fast investigations of the BMD distribution for different implant positions possible. The evaluation of the BMD for a new implant position requires solely the evaluation of $\varvec{w}(\varvec{\mu })$ in Equation (26). Hence, the computational time is reduced by a factor in the magnitude of $10^{6}$ with an overall MAE of 0.005 compared to the evaluation using the high-fidelity model. Additionally, the POD-RBF approximation is more efficient with respect to the required hardware resources, as only the RBF interpolator needs to be evaluated for the parameter dependency and multiplied with the precomputed POD modes. Hence, the POD-RBF surrogate model can be evaluated on smaller devices like tablets. The reduction in time and resources paves the way for integration of the tool in clinical practice to optimise the implantation outcome for individual patients.

Localised maxima and a larger average error are present in Gruen zone 7. The accuracy could be further increased by including more POD modes in the set-up of the surrogate model, which would slightly increase the computational time. To reduce the computational time in the offline phase, the computation of the snapshots could be easily parallelised.

By using a mesh morphing technique, the tedious and time-consuming remeshing process for new implant positions can be avoided, mitigating potential issues during the meshing procedure. Additionally, the joint load is directly transferred to the morphed nodes of the implant without any pre-processing step. Further, the number of nodes is maintained, which makes the usage of MOR techniques straightforward as the BMD distributions are easily stored in the snapshot matrix without the need for projection. In addition, no projection is needed in the online phase, as the mesh morphing can be used again. For clinical applications, this process could be sped up by using a surrogate model for the mesh creation. Of course, the solution could be visualised directly on the reference mesh without requiring additional post-processing, but it might be less accessible to physicians.

The non-intrusive model offers several advantages. Firstly, commercial FEM software can be used, making it more accessible to physicians. In this work, an Abaqus UEL is used for the bone remodelling. Secondly, the non-intrusive model solely depends on the snapshot data and parameters rather than a specific solver. As a result, solutions from various FEM solvers and even medical imaging data can be incorporated into the snapshot generation process for setting up the surrogate model. However, these results would require projection to the reference mesh.

The parameter intervals of the implant position have been chosen to show the functionality of the POD-RBF surrogate model for hip implant positioning. For application purposes, the parameter intervals for the implant position require adjustment to ensure the limits represent physiological bounds that are feasible for implant positions. This extension requires a close discussion between engineers and physicians. In addition, if a larger parameter space is considered, a different morphing technique, such as applying Laplacian smoothing (Grassi et al. 2011), might be required. When adjusting the parameter range, the influence of the parameters could change and needs to be reassessed in a sensitivity analysis.

A possible extension of the model would be to include the implant size in the surrogate model, which has been included in the morphing algorithm by Zheng et al. (2023). However, including more parameters would considerably increase the computation time in the offline phase and is outside the scope of the current work.

## Conclusions

The proposed non-intrusive POD-RBF surrogate model paves the way for utilisation in clinical practice for rapid evaluation of implant stability. The change in the BMD distribution following implantation can be evaluated for different implant positions. The surrogate model significantly decreases the computational effort for new parameter points and exhibits a low MAE for the predicted BMD distribution compared to the high-fidelity solution. The surrogate model shows promise for optimisation studies to determine optimal implant positions, with potential future adaptations to accommodate inter-patient variability.
